# Genetic structure of *Plasmodium vivax* using the merozoite surface protein 1 *icb5*-6 fragment reveals new hybrid haplotypes in southern Mexico

**DOI:** 10.1186/1475-2875-13-35

**Published:** 2014-01-29

**Authors:** René Cerritos, Lilia González-Cerón, José A Nettel, Ana Wegier

**Affiliations:** 1 Departamento de Medicina Experimental, Facultad de Medicina, Universidad Nacional Autónoma de México, Apartado postal 70-275, México D.F. 04510, México; 2 Centro Regional de Investigación en Salud Pública, Instituto Nacional de Salud Pública, Tapachula, Chiapas, México; 3 CENID-COMEF, Instituto Nacional Investigaciones Forestales, Agrícolas y Pecuarias México, Progreso 5, D.F. 04010, Barrio Santa Catarina, Coyoacán, México

**Keywords:** *Plasmodium vivax*, Mexico, Malaria, Merozoite surface protein 1, Haplotype network, Population genetic, Genetic diversity, Recombination analysis

## Abstract

**Background:**

*Plasmodium vivax* is a protozoan parasite with an extensive worldwide distribution, being highly prevalent in Asia as well as in Mesoamerica and South America. In southern Mexico, *P. vivax* transmission has been endemic and recent studies suggest that these parasites have unique biological and genetic features. The *msp1* gene has shown high rate of nucleotide substitutions, deletions, insertions, and its mosaic structure reveals frequent events of recombination, maybe between highly divergent parasite isolates.

**Methods:**

The nucleotide sequence variation in the polymorphic icb5-6 fragment of the *msp1* gene of Mexican and worldwide isolates was analysed. To understand how genotype diversity arises, disperses and persists in Mexico, the genetic structure and genealogical relationships of local isolates were examined. To identify new sequence hybrids and their evolutionary relationships with other *P. vivax* isolates circulating worldwide two haplotype networks were constructed questioning that two portions of the icb5-6 have different evolutionary history.

**Results:**

Twelve new *msp1* icb5-6 haplotypes of *P. vivax* from Mexico were identified. These nucleotide sequences show mosaic structure comprising three partially conserved and two variable subfragments and resulted into five different sequence types. The variable subfragment sV1 has undergone recombination events and resulted in hybrid sequences and the haplotype network allocated the Mexican haplotypes to three lineages, corresponding to the Sal I and Belem types, and other more divergent group. In contrast, the network from icb5-6 fragment but not sV1 revealed that the Mexican haplotypes belong to two separate lineages, none of which are closely related to Sal I or Belem sequences.

**Conclusions:**

These results suggest that the new hybrid haplotypes from southern Mexico were the result of at least three different recombination events. These rearrangements likely resulted from the recombination between haplotypes of highly divergent lineages that are frequently distributed in South America and Asia and diversified rapidly.

## Background

*Plasmodium vivax* is a parasite with a high prevalence in several countries around the world [1]. It has been proposed that southeast Asia represents the possible site of origin for *P. vivax*[2-4] and a number of individuals were subsequently dispersed along with human population movements and eventually became established in other regions where conditions and resources were very different [5]. The Mesoamerican region is one of the latest locations where *P. vivax* was introduced, with time to most recent common ancestor of 23 ky compared to South America (309 ky) and other regions outside southeast Asia [6]. In these regions, although transmission is endemic, it is necessary to progress toward the elimination of this disease, and population genetics can be an efficient tool for understanding the origin, dispersion and persistence of parasite genotypes [7].

The southern border of Mexico, a hypo-endemic area for *P. vivax*, may display particular parasite populations under constant human, vector and ecological pressures [8-11]. In endemic areas, the presence of multiclonal genotypes greatly increases the probability of recombination during the sexual cycle in mosquitoes, leading to the generation of sporozoites with unique genotypes [12].

Several molecular markers have been used to study the evolutionary dynamics and genetic mechanisms that occur frequently in *P. vivax*. The merozoite surface protein 1 (MSP1) is important for erythrocyte invasion and is encoded by a large gene comprised of conserved, partially conserved and variable regions [13]. Several studies have shown that different *msp1* genes exhibit a mosaic-type structure due to intense recombination events [14-16]. The inter-species conserved fragment (icb) 5 and 6 shows high polymorphism due to intra-allelic recombination, insertions, deletions and further point mutations, generating a wide range of new genotypes [15-22]. This high gene polymorphism has allowed the identification of different genetic forms of the parasite, most of which are unique [23,24] and therefore suitable for molecular discrimination of relapse episodes [13,25], and useful for phylogenetic studies [26].

In this study, the *msp1* gene icb5-6 fragment of *P. vivax* parasites from southern Mexico was analysed to determine parasite diversity and potential hybridization events, with special emphasis on finding haplotypes that have contributed to the occurrence of new hybrids in Mexico and their evolutionary relationships with other isolates circulating worldwide.

## Methods

### *Plasmodium vivax* isolates and DNA extraction

Infected blood samples were obtained from symptomatic patients diagnosed with *P. vivax* via the examination of Giemsa-stained blood smears at the Regional Center for Research in Public Health (CRISP-INSP) in Southern Chiapas, Mexico during 2002–2003. After informed written consent was obtained, the patients donated five ml of venous blood, and all patients received anti-malarial treatment following Mexican Treatment Guidelines (NOM-032-SSA2-2002) [27,28]. Total DNA was obtained from infected bloods using the QIAamp® DNA Blood Mini kit, according to the manufacturer’s instructions. A 100 μl aliquot of infected blood rendered 200 μl of DNA solution, which was stored at -20°C until use. To screen for mixed genotype infections (vk210 and vk247), the circumsporozoite gene central repeat genotype (*cspr*) was determined by polymerase chain reaction and restriction fragment length polymorphism (PCR-RFLP) as described previously [29]. Among the 106 examined samples, 95 presented single-genotype infections (34 were *cspr* vk210, and 61 were vk247).

**Figure 1:**
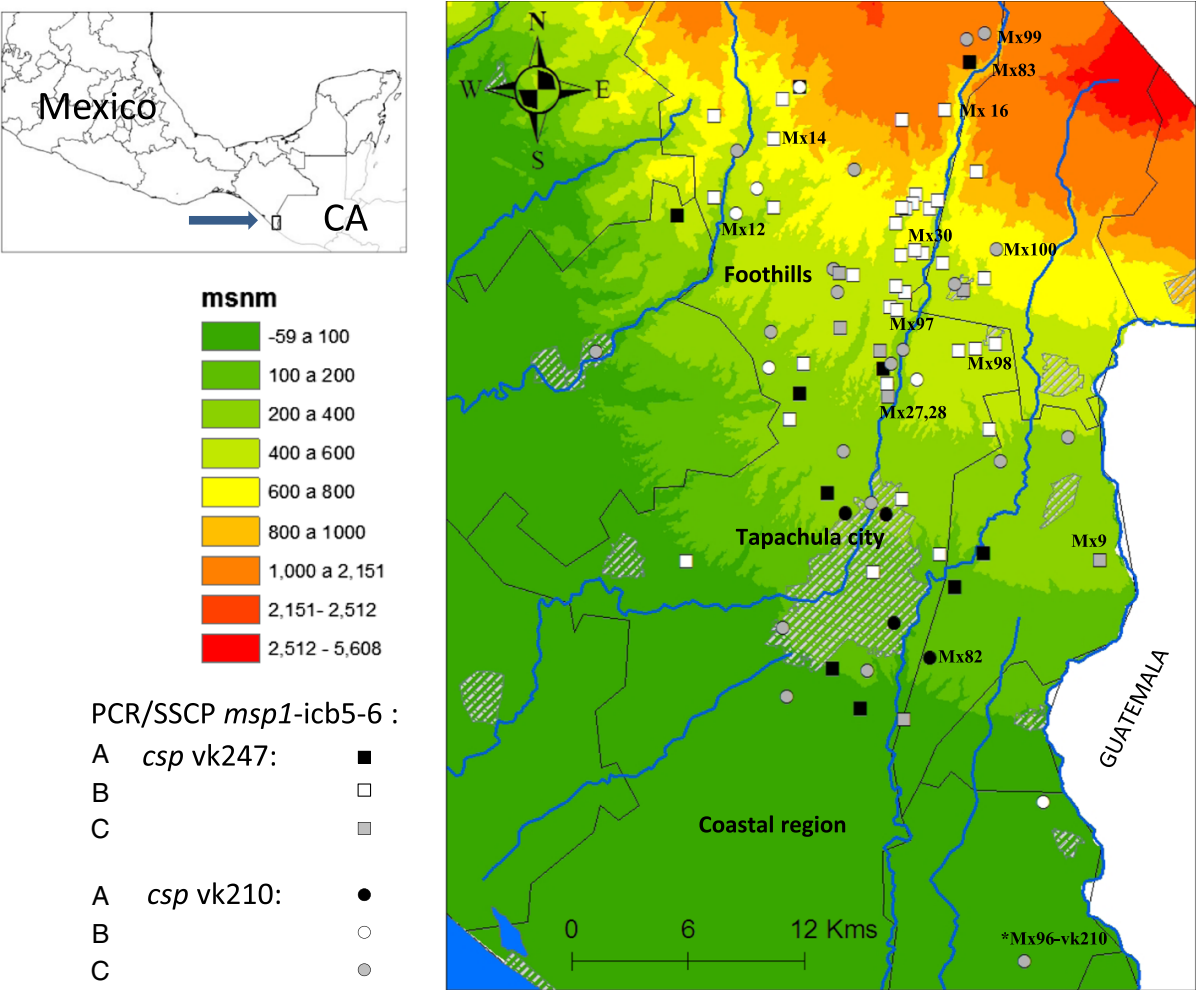
**Study region of southern Mexico.** The site of parasite collection by their *csp-msp1* genotypes is indicated.

### Polymerase chain reaction and single-strand conformational polymorphism (PCR-SSCP) of *msp1* icb5-6 gene fragment

Only single-*cspr* genotype infections were examined regarding the *msp1* icb5-6 polymorphic fragment by PCR-SSCP. Briefly, a nested PCR was carried out. The first PCR amplification contained a mixture of 2 μl of whole DNA from *P. vivax*-infected blood, 4 μl of an oligonucleotide mixture (10 nM), 0.25 μl of each primer (50 pM) Pv200-A5 (5′-tac tac ttg atg gtc ctc- 3′) and Pv200-A6 (5′-cct tct ggt aca ggt aca gct caa tg- 3′) [19], 3 μl of 50 mM MgCl_2_ (Promega), 125 μmol of each deoxynucleotide triphosphate in 1X buffer (100 mM KCl, 20 mM Tris–HCl, pH 7.5) and 0.625 U of Taq DNA Polymerase, in a final volume of 24 μl. The following PCR program was run in a DNA Thermal Cycler (480 PERKIN ELMER, Applied Biosystem CA 94404 USA): 95°C for 5 min, followed by 35 cycles of 95°C for 60 sec, 55°C for 60 sec and 72°C for 60 sec, with a final extension of 72°C for 7 min. The second amplification was performed in a final volume of 50 μl containing 8 μl of 10 nM dNTPs, 5 μl of PCR buffer (10X), 3 μl of 50 mM MgCl_2_, 50 pM oligonucleotides, 0.25 μl of each primer (50 pM) Pv200-1 (5′-cca ctg aga agg cca agc c- 3′), 0.25 μl of Pv200-2 (5′-cca ctg aga aga aca agc c-3′) and 0.5 μl of Pv200-3 (5′-aca ttg aat agg agg tcc a- 3′) [25,30], 0.25 μl of 5 U/μl Taq DNA polymerase (Promega Corporation, Madison USA) and 2 μl of the first-round PCR product. The PCR conditions for the second reaction were the same as above. PCR products of the expected molecular size (600–700 bp) were visualized under UV light following 1% agarose gel electrophoreses and ethidium bromide staining. Then, 50 μl of each PCR product was resolved through electrophoresis in a 1% low melting point agarose gel (Nusieve GTG agarose Invitrogen, Carlsbad, CA) at 115 V for 30 min. The resultant PCR bands were cut out and purified using a gel extraction kit following the manufacturer’s instructions (QIAquick gel extraction kit, QIAGEN, Germany) to recover 30 μl of final volume.

To perform SSCP, a previously reported protocol was used [31]. Briefly, 30 μl of the purified PCR product (from a 50 μl PCR amplification) was denatured by adding loading buffer (1.8 μl of 0.5 M NaOH-10 mM EDTA solution + 1.8 μl of FBB [5% bromophenol blue in formamide] + 1.8 μl of glycerol), followed by heating at 42°C for 5 min. The samples were then immediately quenched on ice and loaded into non-denaturing pre-made polyacrylamide gels (10-well, 8x8 cm, 1.0 mm, 10% TBE gels; Invitrogen Carlsbad CA. USA). Additionally, 15 μl of a 123 bp molecular weight marker (Invitrogen life technologies, Carlsbad, CA, USA) was prepared in glycerol loading buffer (4 μl marker + 5 μl 6X GLB (0.25% bromophenol blue and 30% glycerol in DD water) + 3 μl glycerol + 18 μl water) and loaded into flanking wells. Electrophoresis was performed in 1X glycerol tolerant buffer (20X, 1.78 M Tris ultrapure, 0.57 M Taurina ultrapure and 0.01 M EDTA-Na2-H2O) (USB 71949) at 4°C at a constant voltage of 80 V for 24–126 h. The resultant ssDNA bands were visualized via silver staining [32] and photographed using the BioDoc-it™ digital photo-documentation system (UVP Inc, Upland, California, USA).

### *Msp1* icb5-6 DNA sequencing

Groups of samples showing any of the three observed SSCP patterns were cloned and sequenced. First-round PCR products (Pv200-A5 and Pv200-A6) were cloned using the TOPO TA Cloning® Kit (pCR® 2.1-TOPO® vector from Invitrogen Life Technologies, Carlsbad, CA. USA). Clones of the expected molecular size (600–700 bp) were purified with the QIAprep Spin Miniprep Kit (QIAGEN Germany). At least three different transformed colonies containing the cloned product from each isolate were sequenced using both forward and reverse primers in an ABI PRISM® 3100 Genetic Analyser at the National Institute for Public Health in Cuernavaca, Morelos, Mexico. The electropherogram quality was analysed for each DNA sequence obtained from the Mexican samples using both Phred-Phrap-Consed [33] and via manual inspection, employing the forward and reverse primers as reference. Consensus was achieved using BioEdit (v.4.0), and the nucleotide sequences were deposited at the GenBank NBCI database under accession numbers [GenBank:JX443532- JX443545].

### Data analysis

#### Nucleotide sequences of the icb5-6 fragment

To determine sequence types, the nucleotide sequences obtained from the Mexican parasites were aligned with those corresponding to the Sal I (XM_001614792) [34] and Belem strains (AF435594) [16]. Most parts of this fragment align with either of these sequences.

To carry out the genetic diversity, selection and recombination analyses and to depict the genealogical relationships between Mexican and worldwide parasites, nucleotide sequences with the same gene fragment as those obtained in this study were extracted from the GenBank: Thailand (n = 105: AF435595-AF435615, D85246-D85250, D85252, D85253, D85256-D85259, D85262-D85265, DQ220742, GQ890884, GQ890886, GQ890898, GQ890914, GQ890919, GQ890953, GQ890960, GQ890965, GQ890975, GQ890977, GQ890978 , GQ89098-GQ890998, GQ891000, GQ891003, GQ891005- GQ891041), Brazil (n = 10: AF435593, AF435622, AF435623- AF435625, AF435627, AF435629-AF435631 and Belem strain), India (n = 6: AF435639, AJ250728, AJ494994-AJ494997), Bangladesh (n = 5: AF435616- AF435618, AF435619, AF435620), Turkey (n = 10: AB564562, AB564576, AB564577, AJ494826- AJ494832), Sri Lanka (AJ494998), Mozambique (AJ494999), Italy (AJ250729), Vanuatu (AF435632) and El Salvador (Sal I strain).

### Genetic diversity and selection

The number and frequency of all *msp1* icb5-6 haplotypes and the estimation of the nucleotide diversity for each subfragment from both the Mexican and global populations were determined, including the average nucleotide diversity per site (π) and the expected variation by site under the assumption of neutral evolution (θ). The departure from neutrality with the number of synonymous substitutions per synonymous site, and that of synonymous substitutions per non-synonymous site (dN-dS) were tested using Nei and Gojobori’s method [35,36] with MEGA 2.1 [37].

### Recombination and genealogical relationships

To determine the degree of recombination within and between parasite populations, the minimal number of recombination events (Rm) was estimated by the four gametes test using DnaSP v5 [38]. Historical patterns can be inferred using several different approaches, including examination of haplotype networks that connect each particular haplotype through mutational steps and enable the assignment of extant haplotypes to an ancestral population, making possible to recognize between different processes such as hybridization events from ancestral polymorphisms or migration [39-42]. Therefore to investigate the evolutionary history and relationships between the haplotypes identified in this study, a minimum spanning haplotype network was constructed using TCS 1.21 [43,44]. The *msp1* gene has a mosaic structure [16] and each mosaic piece could depict different evolutionary history. Also, sV1 display three main sequence types, Sal I-like, Belem-like and the hybrid (Sal I-Belem), this hybrid has been reported for parasites in different locations studied so far [12,16-18,22,45-47]. Based on this evidence, the sequence of fragment icb5-6 without sV1 and sV1 alone were analysed separately as the whole fragment would probably show a non-existing genealogy. The method described by Templeton and Sing was used to break any loops (ambiguous connections) present within the network [48], employing predictions derived from coalescence theory [49]. For the construction of two haplotype networks all polymorphic sites that showed higher recombination signal and the poly-Q domain were excluded estimating linkage disequilibrium between polymorphic sites using DnaSP v5 [38]. To root the network and allow ancestral haplotypes to be proposed, the sequence of *Plasmodium cynomolgi* (U25743) was included in the analysis.

### Ethical clearance

This study was approved by the Ethics Committee of the National Institute of Public Health (INSP).

## Results

### *Msp1* icb5-6 SSCP genotype frequency and circumsporozoite repeat type

The PCR-SSCP analysis of the *msp*1 icb5-6 fragments obtained from single-*P. vivax* genotype infections allowed three patterns to be distinguished, designated as A, B and C (see Additional file 1). PCR-SSCP pattern A (n = 20, 21.0%; four vk210 and 16 vk247); pattern B was the most frequent (n = 43, 45.3%; six vk210 and 37 vk247); and pattern C (n = 30, 32.6%; seven vk247 and 23 vk210) (Figure 1). No SSCP results were obtained for two other samples, one of which was isolate Mx96 (vk210) from the coastal region, where a much lower transmission rate is observed than in the foothills.

### Sequence types of the *msp1* icb5-6 gene fragment

**Figure 2:**
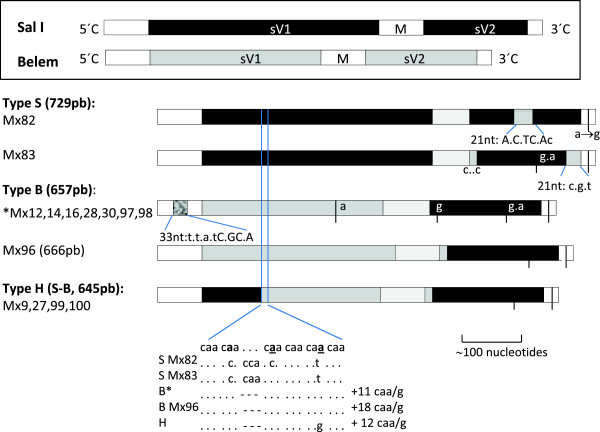
**Nucleotide sequence types of the *P. vivax msp1 *icb5-6 polymorphic fragment of Mexican isolates.** Five different sequence types were constructed according to its homology to the reference strains: gene fragment of Sal I (XM001614792; 2035–2760 nt) and of Belem (AF435594; 2047–2709 nt) strains. Blank boxes are partially conserved subfragments. Black and grey boxes are polymorphic subfragments. Eight-linked nucleotide changes within 33 nt were conserved in 5′C end of sequence type B. Belem domains (compared to Sal I sequence, three and six linked changes within 21 nt each at sV2 in isolates Mx82 and mx83, respectively). Other common nucleotide changes are indicated with dashes and letters. Based on subfragment sV1, three main sequence types are indicated: B (Belem), S (Sal I) and H (Sal I-Belem). nt, nucleotide.

A total of fourteen sequences ranging from 645 to 729 bp were obtained from single *P. vivax* genotype infections, they comprised codons 678–920 and 683–903 of the Sal I and Belem sequences. To carry out a detailed nucleotide analysis, the icb5-6 gene fragment was divided into five subfragments resulted from the alignment of Mexican, Sal I and Belem sequences, three partially conserved (5′C, M and 3′C) flanking two polymorphic ones (sV1 and sV2) (Figure 2; see Additional file 2). The 5′C end comprises 84 bp; the middle (M) is composed of 69 bp and 3′C end consists of 24 pb. Subfragments 5′C and M flank the polymorphic sV1 subfragment; while M and 3′C flank subfragment sV2 (180 bp) (see Additional file 2).

Common polymorphisms were detected among Mexican sequences (see Additional file 2). A pair of non-synonymous changes was detected in the sV2 subfragment of the Sal I sequence, at codons 879 N (aac) → Lys (aag) and 881 Thr (act) → Asn (aat). The change at codon 879 was not present in isolate Mx16. Similarly, at the 3′C end, all of the Mexican parasites exhibited a non-synonymous nucleotide change at codon 920 Lys (aag) → Arg (agg). Based on the sV1 subfragment type, icb5-6 fragment has been clustered into the three basic sequence types: Belem type (B), Sal I type (S) and the Hybrid (H) (Sal I-Belem) [16], as was also observed in Mexican parasites (Figure 2). Further examination of insertions, deletions and nucleotide changes in icb5-6 fragment yielded 12 genotypes (see Additional file 2).

Sequence type S (sV1-Sal I) was detected in isolates Mx82 and Mx83 from the 20 isolates showing SSCP pattern A, which consisted of 729 bp and displayed high similarity to the Sal I sequence. Both isolates showed an insertion between codons 745–746; caa (Gln) and cca (Phe), respectively (Figure 2; see Additional file 2). In addition, subfragment sV1 had a non-synonymous change at codon 798 Ile (att) → Thr (act). The sV2 subfragment from Mx82 was similar to Sal I, except of seven codons 858–864 that had 6 linked nucleotide changes that aligned to the Belem sequence. While in Mx83, the first two codons of sV2 were atc-ttc and three-linked nucleotide changes (portion comprising codons 888–895) aligned to Belem sequence.

Sequence type B (sV1-Belem) was detected in isolates (Mx12, Mx14, Mx16, Mx28, Mx30, Mx97 and Mx98) with SSCP pattern B. All of these sequences comprise 657 nucleotides and have three main characteristics: (1) Alike Belem and Sal I sequences, had eight linked nucleotide changes scattered within 11 codons at the 5′C end. According to the Belem sequence, they had four synonymous nucleotide changes at codons 697 gac → gat, 698 ttc → ttt, 700 ccc → cca and 702 atc → att, and four non-synonymous changes producing three amino acid substitutions at codons 703 Ser (gag) → Ala (cag), 704 Glu (agc) → Gln (gcc) and 707 Ala (gcc) → Thr (acc) (see Additional file 2). (2) The sV1 subfragment contained 17 Gln residues (15 encoded by caa and two encoded by cag). A non-synonymous nucleotide change was observed at codon 794 Gly (ggc) → Asp (gac). (3) In contrast, the sV2 sequence displayed a high similarity to the Sal I strain, and according to this sequence, had a synonymous change at codon 855 gta → gtg (see Additional file 2).

There was additional micro-heterogeneity observed in the nucleotide sequences. The Mx28 isolate showed three non-synonymous nucleotide changes: one in sV1 at the post-Gln repeat (Ser (agt) → Gly (ggt)) and two in sV2 (Asn (aac) → His (cac) and Ser (tcc) → Pro (ccc), respectively). In sub-fragment sV2, Mx97 and Mx16 had a synonymous change (gat → gac), and (acc → aat), respectively. No single nucleotide changes in Mx28 and Mx16 were revealed by the SSCP analysis.

Isolate Mx96 also had a sequence type B (SSCP not shown) and comprised 666 nucleotides. Comparing to the Belem sequence, Mx96 had a non-synonymous change at codon 694 Lys (aag) → Arg (acg) at the 5′C end. Subfragment sV1 was similar to the corresponding Belem sequence, containing 24 Gln residues (22 encoded by caa and two by cag). This was the only sequence presenting a synonymous change in the M subfragment, at codon 817 (gaa → gag). However, next to codons 853 atc-854 ttc of Belem sequence, subfragments sV2 and 3′C aligned to the Sal I sequence. Subfragment sV2 displayed a non-synonymous change Glu (gaa) → Lys (aaa) (codon 885 of the Sal I sequence).

Sequence type H (sV1-hybrid) was detected in four isolates with SSCP pattern C (Mx9, Mx27, Mx99 and Mx100) and comprised 645 nucleotides. The initial 37 codons of sV1 aligned to the Sal I sequence, and from the poly-Gln domain forward to Belem sequence (with 18 Gln residues 14 encoded by caa and four by cag, until the first two codons of subfragment sV2 (atc-ttc); most likely Belem sequence). Further downstream, in sV2, Mx27 and Mx9 showed synonymous changes at codons tct → tcc and gaa → gag, respectively.

### Genetic structure

The diversity analysis revealed a large number of genotypes, both among the Mexican parasites and at the worldwide level. Table 1 presents different measures of diversity determined for each gene subfragment. In the 5′C and 3′C ends, three and four different haplotypes were detected in the Mexican and worldwide sequences, respectively. One of the haplotypes for each sequence was exclusive to Mexico. Subfragment sV2 showed nine and 42 haplotypes in Mexico and at the worldwide level, respectively, while sV1 displayed six and 31. Subfragment sV1 presented at least 10 insertion or deletion events in the Mexican parasites and 29 at the worldwide level. All of the insertion and deletion events involve the caa triplet in isolate Mx83 and the aca triplet in Mx96. The dN-dS test analysis of the 155 sequences indicated that the majority of the polymorphic sites in sV1 are under positive selection and it also suggested that other subfragment sequences might be subjected to purifying selection. For the Mexican isolates, the observed values were similar to those found worldwide in sV1 and sV2 subfragments (see Additional file 3).

**Table 1:** **Diversity, recombination and selection parameters for the** ***P. vivax msp1*** **icb5-6 fragment of Mexican and worldwide isolates**

**Subfragment:**	**5′C**	**sV1**	**M**	**sV2**	**3′C**	**Total**
**Worldwide**						
H	3	31	11	45	4	68
Hd	0.187	0.852	0.294	0.865	0.577	0.879
π	0.015	0.087	0.011	0.060	0.027	0.058
ϴ	0.023	0.050	0.029	0.040	0.021	0.039
INDELs	0	29	0	0	0	29
Rm	0	2	1	5	0	11
**Mexico**						
H	3	6	3	9	1	10
Hd	0.604	0.769	0.385	0.912	0	0.923
π	0.054	0.093	0.006	0.019	0	0.055
ϴ	0.034	0.083	0.009	0.026	0	0.050
INDELs	0	10	0	0	0	10
Rm	0	0	0	0	-	2

Mexican and other 155 worldwide sequences are included. H, haplotypes; Hd, haplotype diversity; π and ϴ, nucleotide diversity; INDELs, insertions/deletions; Rm, recombination events.

Recombination signals were found in Mexican and worldwide clusters, according to different indices. Rm analyses showed two in the Mexican fragments and 11 recombinant sites in the worldwide sequences. Similarly, analysis of linkage disequilibrium detected a high proportion of non-random associations within and between positions in the sV1 and sV2. In general, these analyses indicated that the evolutionary dynamics are not different in these two clusters; in both worldwide and Mexican samples, subfragment sV1, and possibly sV2, are under positive selection while 3′C has experienced negative selection.

### Genealogical haplotype network of *msp1* icb5-6

Fifty-three worldwide haplotypes were obtained for the icb5-6 fragment. Thailand was the country displaying the greatest number of haplotypes (n = 25), followed by Mexico with 12 new haplotypes reported in here. Of the 53 worldwide haplotypes, only six were found in more than one geographic region or country, and all six of these haplotypes were isolated in Thailand. The other 47 haplotypes were unique to a single geographic origin.

**Figure 3:**
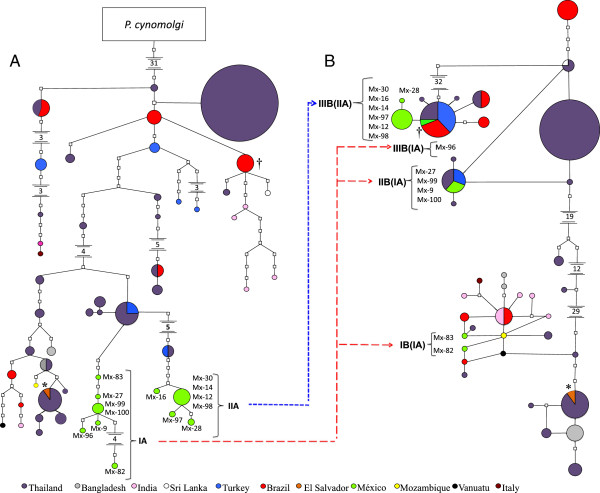
**The haplotype network shows the hybrid nature of the *P. vivax *from southern Mexico.** Two networks were constructed with 155 sequences worldwide, including the 14 Mexican sequences, according to the hypothesis that specific gene fragments based on the icb5-6 sequence correspond to two different parental lineages. The highly recombinant sites were removed to expose the genealogical relationships of the Mexican isolates. **A)** Complete icb5-6 fragment except sV1, **B)** only subfragment sV1. †Belem strain; *Sal I strain. The sizes of the nodes indicate the frequency of a particular haplotype, and colors represent different countries.

The haplotype network A, constructed with subfragment icb5-6 but not sV1 (5′C, M, sV2 and 3′C) showed that the 12 new variants from Mexico were closer to those from Thailand and Turkey, these diverged from Sal I and Belem sequences (Figure 3A). In fact, two lineages were resolved: IA (isolates Mx82, 83, Mx96 and Mx9, 27, 99, 100) and IIA (isolates: Mx12, 14, 16, 28, 30, 97, 98). Parasites of lineage IA were at least 22 and 15 mutational steps from Belem and Sal I, respectively.

While in haplotype network B constructed with sV1, as expected, the isolates of lineage IA were separated into three lineages: IB, IIB and IIIB (Figure 3B). Lineage IB (Mx82, 83) was 5–6 mutational steps from Sal I and 106 from Belem; lineage IIB (Mx96) was 35 steps from Sal I and 73 from Belem; IIIB was Belem and was 106 steps from Sal I. In fact, the Belem and isolate Mx96 haplotypes were found to be the same. Parasites of lineage IIA was at least 31 and 25 mutational steps from Belem and Sal I and were all grouped in lineage IIIB with one and 107 mutational steps from Belem and Sal I.

These results show that at least three different hybridization events occurred and moulded the haplotype diversity, and they further indicate that all *msp1* icb5-6 sequences of *P. vivax* are the result of recombination events and additional diversification.

## Discussion

To understand the processes of transmission, pathogenesis and drug resistance in this species, it is necessary to determine how these parasites originate and disperse. By using genetic population tools it is possible to determine that *P. vivax* presents complex evolutionary dynamics. The *msp1* gene exhibits high genetic variation and complex structuring in a number of geographic regions around the world [16]. Mutation and frequent recombination events are the main mechanisms responsible for the high genetic diversity observed in *P. vivax* icb5-6 fragment [16]. The results from this study resolved not only the icb5-6 nucleotide sequence, which shows a mosaic form in all isolates from Mexico and others around the world, but also the genealogy of possible parental kinship groups. The haplotypes detected in southern Mexican isolates present unique polymorphisms not reported in any other regions. The analysis of the nucleotide sequence of the icb5-6 fragment as well as the relationships of these sequences using the haplotype network clearly demonstrated the hybrid nature of at least three sequence types of the Mexican haplotypes. In a preliminary screening using the PCR-SSCP procedure, at least three different patterns (A, B, C) were obtained in 93 *P. vivax* isolates, and with isolate mx96 comprised 14 nucleotide sequences and five nucleotide sequence types. Twelve of the 14 sequences examined had unique haplotypes endemic to Mexico [18,50]. On the other hand, Thailand exhibits the greatest number of reported sequences (approximately 100), but only 14 different haplotypes were observed, the most abundant was 30% of the samples analysed [16,51].

The results of the haplotype networks suggest that sV1 subfragment has different evolutionary history than the remaining icb5-6 sequence, which agrees with the mosaic structure described in previous works. For lineage IA, it is very likely that isolates Mx9, Mx27, Mx99 and Mx100 correspond to a parental variant that hybridized two or three times with haplotypes from very distant lineages. In the sV1-haplotype networks, isolates Mx83 and Mx82 are closely related to the Sal I strain and isolate Mx96 is closely related to the Belem strain. The genealogical relationship of the sV1 subfragment of these isolates is closer to a lineage similar to Sal I type, and IIB probably the parental haplotypes genealogically nearest to which recombination events had occurred, while IIIB is identical to the Belem strain.

It is possible that haplotypes of isolates Mx9, Mx27, Mx99 and Mx100 diversify rapidly after recombination, producing an increase in the frequency of this allele in the local parasite population. Over 30% of the isolates from Mexico exhibit this sequence type (H). Isolate Mx96 may also have originated from a parent similar to that of the sequence H, but accompanied by intra-allelic recombination with a parental lineage related to the Belem strain. In fact, the sV1 subfragment of isolate Mx96 and the Belem strain display the same haplotype. In general, lineages IA and IIA seem to be descendants of parasites from Thailand and Turkey.

Lineage IIA, which is entirely composed of isolates showing sequences sV1-Belem type (Mx12, Mx14, Mx16, Mx28, Mx30, Mx97 and Mx98), most likely originated through a single recombination event with lineage IIIB (Belem type). The presence of additional polymorphisms among these isolates indicates that rapid diversification occurred, producing new genotypes; this assumption is supported by its high frequency in the sample (approximately 45.3%), as observed in other related parasites [52,53].

It is important to mention that hypothetical parental groups were not detected in Mexico, i.e., that of lineage IIIB, that gave rise to subfragment sV1 (Belem type). Two possible explanations for this finding are 1) that the frequency of these haplotypes in the population is very low and 2) that there is a lack of ancestral haplotypes due to the migration of only descendant-recombinant forms, as has been detected in some regions, such as Korea [45]. Moreover, the frequent occurrence of recombination and hybridization events can be explained by the existence of multi-genotype infections in individuals living in endemic areas for malaria [7]. One host may exhibits two or more genetic variants, and recombination events are likely to occur in a such way that genetic variation at the population level is increased due to these co-infections. The haplotypes found in Mexico seem to have originated in this manner from two parental lineages that were very distant phylogenetically. These ancestral haplotypes gave rise to three or four hybrid groups (also by recombination) that may persist currently because they are being positively selected [54,55].

In fact, haplotypes found in Mexico display two different histories: we found exclusive clades in this region appearing to be the result of historical migrations that diverged and diversified over time, with no evidence that *P. vivax* migrated from Mexico to other sites, while the recent migration events identified from Southeast Asia indicate that the probability of finding haplotypes that originated elsewhere is high [2], from Middle East (Turkey) and/or Thailand. Accordingly, *pvs25* and *pvs28*[9], and the *csp*[56] genes of *P. vivax* from southern Mexico were found close related to parasites from Iran and Eastern Asia and probably much of the gene pool of Mexico has been provided by genotypes of these countries that have suffered continuously migration, which needs further investigation.

The twelve novel Mexican haplotypes were likely the result of recombination events which are maintained by local selective pressures. The relevance of these results lies in the possibility of depicting and predicting potential dispersal events and understanding how new epidemic outbreaks are caused by this parasite. Further extensive analyses performed in Mexico and Mesoamerica, and the use of nuclear and mitochondrial markers will help to reveal events of *P. vivax* migration and dispersal, which could be relevant for understanding the infectious process of this species.

## Conclusions

The diversification and range expansion observed in the Mexican haplotypes were consistent with previous reports, as the genealogy suggests that haplotypes from Central America, Europe, Africa and other regions of Southeast Asia derived from one ancestral group from Thailand. Based on the genealogical analysis of the *msp1* gene icb5-6 fragment, it is demonstrated not only the diversification process of the haplotypes originated in Mexico, but it is likely that these haplotypes are the emerging product of recombination events between genealogically separated lineages found in Thailand and Turkey. Careful analyses of the evolutionary history of *P. vivax* can contribute to design global health policies to treat this parasite. Even greater sampling efforts are needed to understand this history, but the information provided in this study will be useful and will encourage further discussions and questions. In the future it will be necessary to determine whether local adaptations are linked to the hosts using other markers and new tools for data analyses.

## Competing interests

The authors declare that they have no competing interests.

## Authors’ contributions

RC carried out the genetic analysis, organized information in figures and tables, interpreted data and drafted the manuscript. AW with RC conceived the genealogy haplotype network analysis. AW also organized information in figures and tables, interpreted data and drafted the manuscript. LGC participated in the study design and data analysis, preparation of figures and interpretation of data, and drafted the manuscript. JN participated in PCR-SSCP analysis, PCR amplification and gene cloning, nucleotide sequence alignment. All authors read and approved the final manuscript.

## Supplementary Material

Additional file 1 ***P. vivax msp1 *** **icb5-6 gene fragment amplified by PCR and single-strand conformational polymorphism (SSCP) analyses.** A) Agarose gel (lanes 1–16) showing the molecular size of the amplified PCR product. Cn, control negative. B) Acrylamide gel showing the three SSCP patterns observed in the Mexican isolates. SSCP-A (lanes 4, 5 and 7), SSCP-B (lanes 2, and 6), SSCP-C (lanes 1 and 8). M: molecular size markers of 100 Kb. The SSCP pattern of isolate Mx96 was not obtained. Click here for file

Additional file 2 **Nucleotide and amino acid polymorphism of the *msp1 *** **icb5-6 gene fragment of 14 *P. vivax *** **isolates from Mexico.** The nucleotide sequences were aligned to the reference sequences of Belem (AF435594; codons 683–903) and Sal I (XM_001614792; codons 707–920) strains. This indicates the three partially conserved (5′-end, M and 3′end) and two variable (sV1 and sV2) nucleotide subfragments. The isolate codes are provided. Click here for file

Additional file 3 **Neutrality test for *P. vivax msp1 *** **icb5-6 gene fragment.** The test is based in the number of synonymous substitutions per synonymous site, and that of non-synonymous substitutions per non-synonymous site. Subfragments sV1 and sV2 showed an high proportion of polymorphic sites under positive selection. Click here for file
