# Supplementary figures and images for: Genetic structure of Plasmodium vivax using the merozoite surface protein 1 icb5-6 fragment reveals new hybrid haplotypes in southern Mexico
Source: Malar J. 2014 Jan 29;13:35. doi: 10.1186/1475-2875-13-35 (PMC3923247; doi:10.1186/1475-2875-13-35)

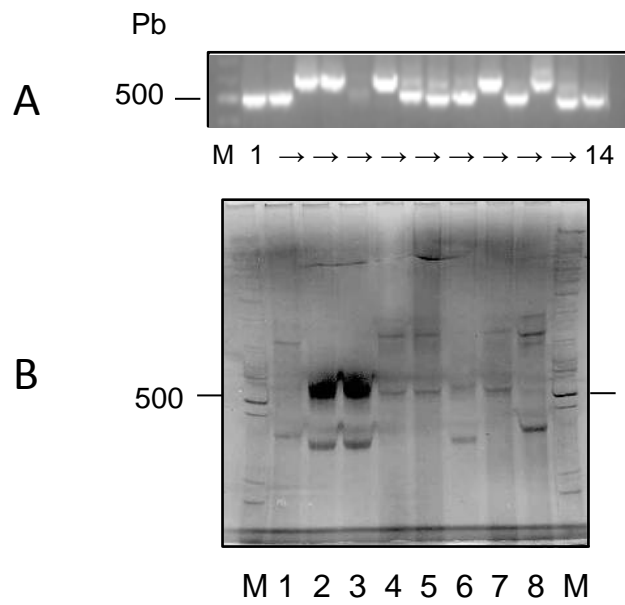

Supplement: Additional file 1 — P. vivax msp1 icb5-6 gene fragment amplified by PCR and single-strand conformational polymorphism (SSCP) analyses. A) Agarose gel (lanes 1–16) showing the molecular size of the amplified PCR product. Cn, control negative. B) Acrylamide gel showing the three SSCP patterns observed in the Mexican isolates. SSCP-A (lanes 4, 5 and 7), SSCP-B (lanes 2, and 6), SSCP-C (lanes 1 and 8). M: molecular size markers of 100 Kb. The SSCP pattern of isolate Mx96 was not obtained. [file 1475-2875-13-35-S1.pdf]

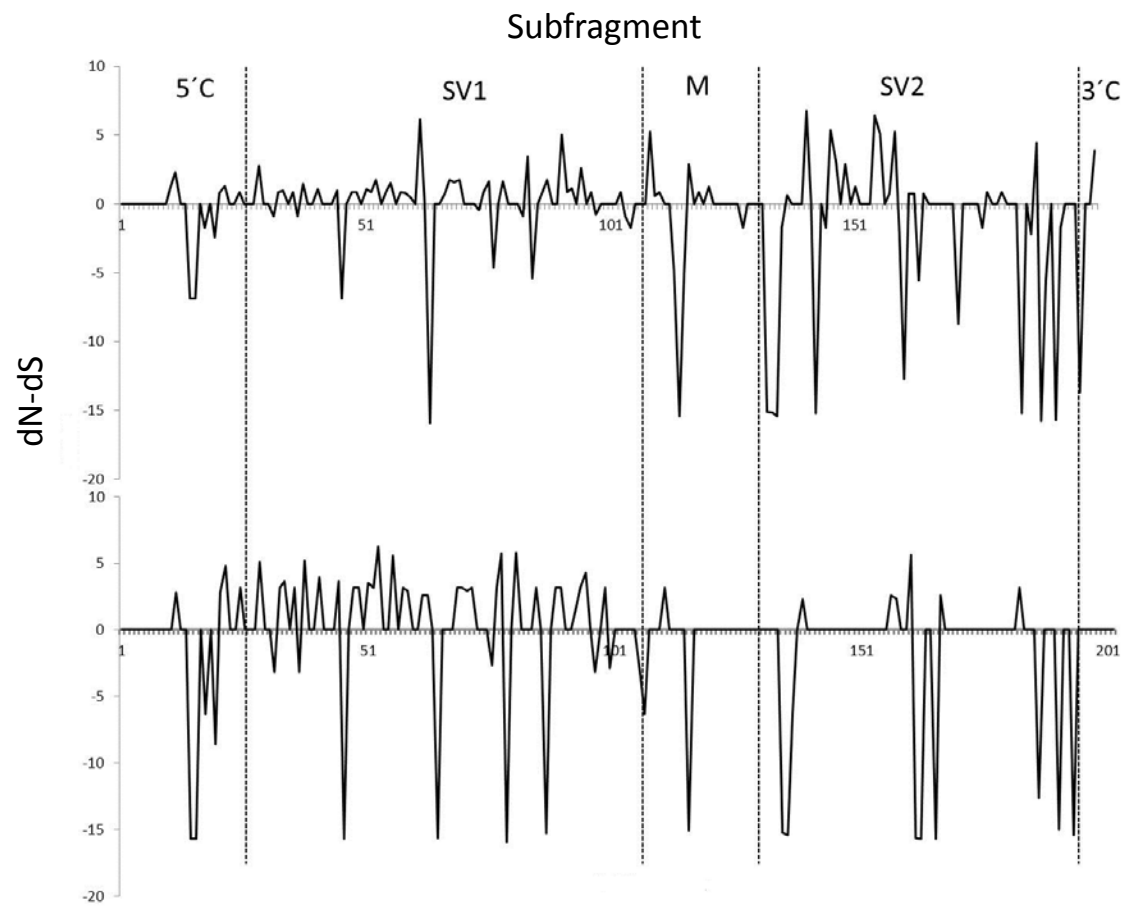

Supplement: Additional file 3 — Neutrality test for P. vivax msp1 icb5-6 gene fragment. The test is based in the number of synonymous substitutions per synonymous site, and that of non-synonymous substitutions per non-synonymous site. Subfragments sV1 and sV2 showed an high proportion of polymorphic sites under positive selection. [file 1475-2875-13-35-S3.pdf]
