# Supplementary material for: Genetic structure of Plasmodium vivax using the merozoite surface protein 1 icb5-6 fragment reveals new hybrid haplotypes in southern Mexico
Source: Malar J. 2014 Jan 29;13:35. doi: 10.1186/1475-2875-13-35 (PMC3923247; doi:10.1186/1475-2875-13-35)
Supplement: Additional file 2 — Nucleotide and amino acid polymorphism of the msp1 icb5-6 gene fragment of 14 P. vivax isolates from Mexico. The nucleotide sequences were aligned to the reference sequences of Belem (AF435594; codons 683–903) and Sal I (XM_001614792; codons 707–920) strains. This indicates the three partially conserved (5′-end, M and 3′end) and two variable (sV1 and sV2) nucleotide subfragments. The isolate codes are provided. [file 1475-2875-13-35-S2.pdf]

Additional file 2

Line 1

```
                  →  semiconserved subfragment 5´C
      AA  683    Y   Y   L   M   V   L   K   R   E   I   D   K   L   K   D   F   I   P   K   I   E   S   M   I   A   T   E   K
Belem: nt 2047  TAC TAC TTG ATG GTC CTC AAA AGG GAA ATT GAC AAG TTG AAG GAC TTC ATC CCC AAA ATC GAG AGC ATG ATC GCC ACT GAG AAG

Mx12            ... ... ... ... ... ... ... ... ... ... ... ... ... ... ..t ..t ... ..a ... ..t C.. GC. ... ... A.. ... ... ...
Mx14            ... ... ... ... ... ... ... ... ... ... ... ... ... ... ..t ..t ... ..a ... ..t C.. GC. ... ... A.. ... ... ...
Mx16            ... ... ... ... ... ... ... ... ... ... ... ... ... ... ..t ..t ... ..a ... ..t C.. GC. ... ... A.. ... ... ...
Mx28            ... ... ... ... ... ... ... ... ... ... ... ... ... ... ..t ..t ... ..a ... ..t C.. GC. ... ... A.. ... ... ...
Mx30            ... ... ... ... ... ... ... ... ... ... ... ... ... ... ..t ..t ... ..a ... ..t C.. GC. ... ... A.. ... ... ...
Mx97            ... ... ... ... ... ... ... ... ... ... ... ... ... ... ..t ..t ... ..a ... ..t C.. GC. ... ... A.. ... ... ...
Mx98            ... ... ... ... ... ... ... ... ... ... ... ... ... ... ..t ..t ... ..a ... ..t C.. GC. ... ... A.. ... ... ...

Mx96            ... ... ... ... ... ... ... ... ... ... ... .G. ... ... ... ... ... ... ... ... ... ... ... ... ... ... ... ...

Mx9             ... ... ... ... ... ... ... ... ... ... ... ... ... ... ... ... ... ... ... ... ... ... ... ... ... ... ... ...
Mx27            ... ... ... ... ... ... ... ... ... ... ... ... ... ... ... ... ... ... ... ... ... ... ... ... ... ... ... ...
Mx99            ... ... ... ... ... ... ... ... ... ... ... ... ... ... ... ... ... ... ... ... ... ... ... ... ... ... ... ...
Mx100           ... ... ... ... ... ... ... ... ... ... ... ... ... ... ... ... ... ... ... ... ... ... ... ... ... ... ... ...
Mx83            ... ... ... ... ... ... ... ... ... ... ... ... ... ... ... ... ... ... ... ... ... ... ... ... ... ... ... ...
Mx82            ... ... ... ... ... ... ... ... ... ... ... ... ... ... ... ... ... ... ... ... ... ... ... ... ... ... ... ...

SalI: nt 2035   ... ... ... ... ... ... ... ... ... ... ... ... ... ... ... ... ... ... ... ... ... ... ... ... ... ... ... ...  28
      AA 678     -   -   -   -   -   -   -   -   -   -   -   R   -   -   -   -   -   -   -   -   Q   A   -   -   T   -   -   -
```

Line 2

```
                 →  sV1 polymorphic subfragment:
      AA 711     A   K   P   A   A   S   A   P   V   T   S   G   Q   L   L   R   G   S   S   E   A   A   T   E   V   T   T   N
Belem: nt 2131  GCC AAG CCG GCA GCG TCA GCG CCA GTG ACC AGT GGA CAA TTG CTT AGA GGA TCA AGC GAA GCA GCG ACA GAG GTC ACA ACC AAT

Mx12            ... ... ... ... ... ... ... ... ... ... ... ... ... ... ... ... ... ... ... ... ... ... ... ... ... ... ... ...
Mx14            ... ... ... ... ... ... ... ... ... ... ... ... ... ... ... ... ... ... ... ... ... ... ... ... ... ... ... ...
Mx16            ... ... ... ... ... ... ... ... ... ... ... ... ... ... ... ... ... ... ... ... ... ... ... ... ... ... ... ...
Mx28            ... ... ... ... ... ... ... ... ... ... ... ... ... ... ... ... ... ... ... ... ... ... ... ... ... ... ... ...
Mx30            ... ... ... ... ... ... ... ... ... ... ... ... ... ... ... ... ... ... ... ... ... ... ... ... ... ... ... ...
Mx97            ... ... ... ... ... ... ... ... ... ... ... ... ... ... ... ... ... ... ... ... ... ... ... ... ... ... ... ...
Mx98            ... ... ... ... ... ... ... ... ... ... ... ... ... ... ... ... ... ... ... ... ... ... ... ... ... ... ... ...
Mx96            ... ... ... ... ... ... ... ... ... ... ... ... ... ... ... ... ... ... ... ... ... ... ... ... ... ... ... ...

Mx9             AA. ... ... A.C .T. G.. ... G.. .AT .TA GTG .C. A.G GGA .AA TCG CTT AG. GGA .C. AGT .AA ... .G. ACA ..t GG. ...
Mx27            AA. ... ... A.C .T. G.. ... G.. .AT .TA GTG .C. A.G GGA .AA TCG CTT AG. GGA .C. AGT .AA ... .G. ACA ..t GG. ...
Mx99            AA. ... ... A.C .T. G.. ... G.. .AT .TA GTG .C. A.G GGA .AA TCG CTT AG. GGA .C. AGT .AA ... .G. ACA ..t GG. ...
Mx100           AA. ... ... A.C .T. G.. ... G.. .AT .TA GTG .C. A.G GGA .AA TCG CTT AG. GGA .C. AGT .AA ... .G. ACA ..t GG. ...
Mx83            AA. ... ... A.C .T. G.. ... G.. .AT .TA GTG .C. A.G GGA .AA TCG CTT AG. GGA .C. AGT .AA ... .G. ACA ..t GG. ...
Mx82            AA. ... ... A.C .T. G.. ... G.. .AT .TA GTG .C. A.G GGA .AA TCG CTT AG. GGA .C. AGT .AA ... .G. ACA ..t GG. ...
SalI: nt 2119   AA. ... ... A.C .T. G.. ... G.. .AT .TA GTG .C. A.G GGA .AA TCG CTT AG. GGA .C. AGT .AA ... .G. ACA ..t GG. ...
      AA 707     N   -   -   T   V   A   -   A   D   I   V   A   K   G   Q   S   L   R   G   A   S   E   -   G   T   -   G   -
```

Line 3

```
                 → sV1 polymorphic subfragment
       AA 739    A   V   T   S   E   V   Q   Q   Q   Q   Q   Q   Q   Q   Q   Q   Q   Q   Q   Q   Q   Q   -/Q Q   Q   Q   Q   Q   Q
Belem: nt 2215   GCG GTA ACA TCT GAA GTA CAA CAA CAA CAA CAA CAA CAA CAA CAA CAA CAA CAA CAA CAA CAA CA- --G CAA CAA CAG CAA CAA CAA

Mx12             ... ... ... ... ... ... ... ... ... ... ... ... ... ... ... ..- --- --- --- --- --- --- --. ... ... ... ... ... ...
Mx14             ... ... ... ... ... ... ... ... ... ... ... ... ... ... ... ..- --- --- --- --- --- --- --. ... ... ... ... ... ...
Mx16             ... ... ... ... ... ... ... ... ... ... ... ... ... ... ... ..- --- --- --- --- --- --- --. ... ... ... ... ... ...
Mx28             ... ... ... ... ... ... ... ... ... ... ... ... ... ... ... ..- --- --- --- --- --- --- --. ... ... ... ... ... ...
Mx30             ... ... ... ... ... ... ... ... ... ... ... ... ... ... ... ..- --- --- --- --- --- --- --. ... ... ... ... ... ...
Mx97             ... ... ... ... ... ... ... ... ... ... ... ... ... ... ... ..- --- --- --- --- --- --- --. ... ... ... ... ... ...
Mx98             ... ... ... ... ... ... ... ... ... ... ... ... ... ... ... ..- --- --- --- --- --- --- --. ... ... ... ... ... ...
Mx96             ... ... ... ... ... ... ... ... ... ... ... ... ... ... ... ... ... ... ... ... ... ..A CA. ... ... ... ... ... ...

Mx9              A.A ..c .AT G.G C.. AC. GCT GT. GT. ... ... ... ... ..g ... ... ..g ... ... ..- --- --- --. ... ... ... ... ... ...
Mx27             A.A ..c .AT G.G C.. AC. GCT GT. GT. ... ... ... ... ..g ... ... ..g ... ... ..- --- --- --. ... ... ... ... ... ...
Mx99             A.A ..c .AT G.G C.. AC. GCT GT. GT. ... ... ... ... ..g ... ... ..g ... ... ..- --- --- --. ... ... ... ... ... ...
Mx100            A.A ..c .AT G.G C.. AC. GCT GT. GT. ... ... ... ... ..g ... ... ..g ... ... ..- --- --- --. ... ... ... ... ... ...

Mx83             A.A ..c .AT G.G C.. AC. GCT GT. GT. ... .C. .C. ... ..T ... GT. GT. A.T GC. GT. ACG GTA CA. .CT GG. ACA AC. GG. ..T
Mx82             A.A ..c .AT G.G C.. AC. GCT GT. GT. ... .C. ... ... ..T ... GT. GC. A.T GC. GT. ACG GTA CA. .CT GG. ACA AC. GG. ..T
SalI: nt 2203    A.A ..c .AT G.G C.. AC. GCT GT. GT. ... .C. --- ... ..T ... GT. GT. A.T GC. GT. ACG GTA CA. .CT GG. ACA AC. GG. ..T
      AA 735     T   V   N   A   Q   T   A   V   V   -   P   P   -   H   -   V   V/A N   A   V   T   V   Q   P   G   T   T   G   H
```

Line 4

```
                 → sV1 polymorphic subfragment

       AA 767    Q   -   -   -   -   -   -   -   -   -   -   -   -   -   -   -   S   Q   V   V   P   A   P   A   G   D   A   Q
Belem: nt 2299   CAA --- --- --- --- --- --- --- --- --- --- --- --- --- --- --- TCA CAA GTA GTA CCA GCA CCT GCA GGA GAT GCC CAA

Mx12             ... --- --- --- --- --- --- --- --- --- --- --- --- --- --- --- ... ... ... ... ... ... ... ... ... ... ... ...
Mx14             ... --- --- --- --- --- --- --- --- --- --- --- --- --- --- --- ... ... ... ... ... ... ... ... ... ... ... ...
Mx16             ... --- --- --- --- --- --- --- --- --- --- --- --- --- --- --- ... ... ... ... ... ... ... ... ... ... ... ...
Mx28             ... --- --- --- --- --- --- --- --- --- --- --- --- --- --- --- ... ... ... ... ... ... ... ... ... ... ... ...
Mx30             ... --- --- --- --- --- --- --- --- --- --- --- --- --- --- --- ... ... ... ... ... ... ... ... ... ... ... ...
Mx97             ... --- --- --- --- --- --- --- --- --- --- --- --- --- --- --- ... ... ... ... ... ... ... ... ... ... ... ...
Mx98             ... --- --- --- --- --- --- --- --- --- --- --- --- --- --- --- ... ... ... ... ... ... ... ... ... ... ... ...
Mx96             ... --- --- --- --- --- --- --- --- --- --- --- --- --- --- --- ... ... ... ... ... ... ... ... ... ... ... ...

Mx9              ... --- --- --- --- --- --- --- --- --- --- --- --- --- --- --- ... ... ... ... ... ... ... ... ... ... ... ...
Mx27             ... --- --- --- --- --- --- --- --- --- --- --- --- --- --- --- ... ... ... ... ... ... ... ... ... ... ... ...
Mx99             ... --- --- --- --- --- --- --- --- --- --- --- --- --- --- --- ... ... ... ... ... ... ... ... ... ... ... ...
Mx100            ... --- --- --- --- --- --- --- --- --- --- --- --- --- --- --- ... ... ... ... ... ... ... ... ... ... ... ...

Mx83             ... GCA CAA GGT GGA GAA GCA GAA ACA CAA ACA AAT TCA GTA CAA GCA G.. ... ..t CA. .A. A.. ... ... ... .CG .G. GG.
Mx82             ... GCA CAA GGT GGA GAA GCA GAA ACA CAA ACA AAT TCA GTA CAA GCA G.. ... ..t CA. .A. A.. ... ... ... .CG .G. GG.

SalI: nt 2287    ... GCA CAA GGT GGA GAA GCA GAA ACA CAA ACA AAT TCA GTA CAA GCA G.. ... ..t CA. .A. A.. ... ... ... .CG .G. GG.
       AA 763    -   A   Q   G   G   E   A   E   T   Q   T   N   S   V   Q   A   A   -   -   Q   Q   T   -   -   -   A   G   G
```

Line 5

```
                  → sV1 polymorphic subfragment:
      AA 780     Q   V   I   S   T   Q   P   T  S/G  Q   S   A   A   P  G/D  V   -   S   A   T   P   A   P   -   -   -   -   -   T
Belem: nt 2338  CAA GTA ATC TCA ACA CAA CCG ACT AGT CAA TCC GCA GCA CCA GGC GTA --- TCA GCC ACA CCA GCA CCA --- --- --- --- --- ACA

Mx30            ... ... ... ... ... ... ... ... ... ... ... ... ... ... .A. ... --- ... ... ... ... ... ... --- --- --- --- --- ...
Mx16            ... ... ... ... ... ... ... ... ... ... ... ... ... ... .A. ... --- ... ... ... ... ... ... --- --- --- --- --- ...
Mx14            ... ... ... ... ... ... ... ... ... ... ... ... ... ... .A. ... --- ... ... ... ... ... ... --- --- --- --- --- ...
Mx97            ... ... ... ... ... ... ... ... ... ... ... ... ... ... .A. ... --- ... ... ... ... ... ... --- --- --- --- --- ...
Mx12            ... ... ... ... ... ... ... ... ... ... ... ... ... ... .A. ... --- ... ... ... ... ... ... --- --- --- --- --- ...
Mx28            ... ... ... ... ... ... ... ... G.. ... ... ... ... ... .A. ... --- ... ... ... ... ... ... --- --- --- --- --- ...
Mx98            ... ... ... ... ... ... ... ... ... ... ... ... ... ... .A. ... --- ... ... ... ... ... ... --- --- --- --- --- ...
Mx-96           ... ... ... ... ... ... ... ... ... ... ... ... ... ... ... ... --- ... ... ... ... ... ... --- --- --- --- --- ...

Mx9             ... ... ... ... ... ... ... ... ... ... ... ... ... ... ... ... --- ... ... ... ... ... ... --- --- --- --- --- ...
Mx27            ... ... ... ... ... ... ... ... ... ... ... ... ... ... ... ... --- ... ... ... ... ... ... --- --- --- --- --- ...
Mx99            ... ... ... ... ... ... ... ... ... ... ... ... ... ... ... ... --- ... ... ... ... ... ... --- --- --- --- --- ...
Mx100           ... ... ... ... ... ... ... ... ... ... ... ... ... ... ... ... --- ... ... ... ... ... ... --- --- --- --- --- ...

Mx83            ..g ... GC. ... ... ... A.. ... ..c ... G.. C.. ... ... ACT CA. GCC ..c C.A GA. ... ... ... GCC GCC CCA CCA TCG ...
Mx82            ..g ... GC. ... ... ... A.. ... ..c ... G.. C.. ... ... ACT CA. GCC ..c C.A GA. ... ... ... GCC GCC CCA CCA TCG ...

SalI: nt 2371   ..g ... GC. ... ... ... A.. .T. ..c ... G.. C.. ... ... ACT CA. GCC ..c C.A GA. ... ... ... GCC GCC CCA CCA TCG ...
     AA 791      -   -   A   -   -   -   T   I   -   -   A   P   -   -   T   Q   A   -   P   E   -   -   -   A   A   P   P   S   -
```

Line 6

```
                  → sV1 polymorphic subfragment      | → subfragment M
      AA 803     P   A   A   A   A   A   P   A   P   A   M   S   K   L   E   Y   L   E   K   L   L   D   F   L   K   S   A   Y
Belem: nt 2407  CCT GCT GCC GCA GCC GCC CCA GCA CCA GCC ATG TCC AAA CTG GAA TAC CTC GAA AAG CTC CTT GAT TTT TTA AAA TCC GCT TAC

Mx30            ... ... ... ... ... ... ... ... ... ... ... ... ... ... ... ... ... ... ... ... ... ... ... ... ... ... ... ...
Mx16            ... ... ... ... ... ... ... ... ... ... ... ... ... ... ... ... ... ... ... ... ... ... ... ... ... ... ... ...
Mx14            ... ... ... ... ... ... ... ... ... ... ... ... ... ... ... ... ... ... ... ... ... ... ... ... ... ... ... ...
Mx97            ... ... ... ... ... ... ... ... ... ... ... ... ... ... ... ... ... ... ... ... ... ... ... ... ... ... ... ...
Mx12            ... ... ... ... ... ... ... ... ... ... ... ... ... ... ... ... ... ... ... ... ... ... ... ... ... ... ... ...
Mx28            ... ... ... ... ... ... ... ... ... ... ... ... ... ... ... ... ... ... ... ... ... ... ... ... ... ... ... ...
Mx98            ... ... ... ... ... ... ... ... ... ... ... ... ... ... ... ... ... ... ... ... ... ... ... ... ... ... ... ...
Mx96            ... ... ... ... ... ... ... ... ... ... ... ... ... ... ..g ... ... ... ... ... ... ... ... ... ... ... ... ...

Mx9             ... ... ... ... ... ... ... ... ... ... ... ... ... ... ... ... ... ... ... ... ... ... ... ... ... ... ... ...
Mx27            ... ... ... ... ... ... ... ... ... ... ... ... ... ... ... ... ... ... ... ... ... ... ... ... ... ... ... ...
Mx99            ... ... ... ... ... ... ... ... ... ... ... ... ... ... ... ... ... ... ... ... ... ... ... ... ... ... ... ...
Mx100           ... ... ... ... ... ... ... ... ... ... ... ... ... ... ... ... ... ... ... ... ... ... ... ... ... ... ... ...

Mx83            ... ... ... ... .TT ..t ... ... ... A.. ... ... ... ... ... ... ... ... ... ... ... ... ... ... ... ... ... ...
Mx82            ... ... ... ... .TT ..t ... ... ... A.. ... ... ... ... ... ... ... ... ... ... ... ... ... ... ... ... ... ...
SalI: nt 2458   ... ... ... ... .TT ..t ... ... ... A.. ... ... ... ... ... ... ... ... ... ... ... ... ... ... ... ... ... ...
     AA 820      -   -   -   -   V   -   -   -   -   T   -   -   -   -   -   -   -   -   -   -   -   -   -   -   -   -   -   -
```

Line 7

→ subfragment M | → sV2 polymorphic subfragment

AA 825	A	C	H	K	H	I	F	V	T	N	S	T	M	D	K	K	L	L	K	E	Y	E	L	N	A	D	E
Belem:nt 2490	**GCA**	**TGT**	**CAC**	**AAG**	**CAC**	**ATC**	**TTC**	**GTA**	**ACC**	**AAC**	**TCC**	**ACC**	**ATG**	**GAC**	**AAG**	**AAA**	**CTA**	**CTC**	**AAA**	**GAG**	**TAC**	**GAA**	**CTT**	**AAC**	**GCT**	**GAT**	**GAG**
Mx96	...	...	...	...	...	...	...	...	...	...	...	...	...	**A.A**	...	**G.G**	...	...	**G.T**	**C..**	...	**A..**	...	...	...	...	...
Mx27	...	...	...	...	...	...	...	...	...	...	...	...	...	**A.A**	...	**G.G**	...	...	**G.T**	**C..**	...	**A..**	...	...	...	...	...
Mx99	...	...	...	...	...	...	...	...	...	...	...	...	...	**A.A**	...	**G.G**	...	...	**G.T**	**C..**	...	**A..**	...	...	...	...	...
Mx9	...	...	...	...	...	...	...	...	...	...	...	...	...	**A.A**	...	**G.G**	...	...	**G.T**	**C..**	...	**A..**	...	...	...	...	...
Mx100	...	...	...	...	...	...	...	...	...	...	...	...	...	**A.A**	...	**G.G**	...	...	**G.T**	**C..**	...	**A..**	...	...	...	...	...
Mx83	...	...	...	...	...	...	...	...	...	...	...	...	...	**A.A**	...	**G.G**	...	...	**G.T**	**C..**	...	**A..**	...	...	...	...	...
Mx30	...	...	...	...	...	**..t**	**..t**	**..g**	...	...	...	...	...	**A.A**	...	**G.G**	...	...	**G.T**	**C..**	...	**A..**	...	...	...	...	...
Mx16	...	...	...	...	...	**..t**	**..t**	**..g**	...	...	...	...	...	**A.A**	...	**G.G**	...	...	**G.T**	**C..**	...	**A..**	...	...	...	...	...
Mx14	...	...	...	...	...	**..t**	**..t**	**..g**	...	...	...	...	...	**A.A**	...	**G.G**	...	...	**G.T**	**C..**	...	**A..**	...	...	...	...	...
Mx97	...	...	...	...	...	**..t**	**..t**	**..g**	...	...	...	...	...	**A.A**	...	**G.G**	...	...	**G.T**	**C..**	...	**A..**	...	...	...	...	...
Mx12	...	...	...	...	...	**..t**	**..t**	**..g**	...	...	...	...	...	**A.A**	...	**G.G**	...	...	**G.T**	**C..**	...	**A..**	...	...	...	...	...
Mx98	...	...	...	...	...	**..t**	**..t**	**..g**	...	...	...	...	...	**A.A**	...	**G.G**	...	...	**G.T**	**C..**	...	**A..**	...	...	...	...	...
Mx28	...	...	...	...	...	**..t**	**..t**	**..g**	...	**C..**	...	...	...	**A.A**	...	**G.G**	...	...	**G.T**	**C..**	...	**A..**	...	...	...	...	...
Mx82	...	...	...	...	...	**..t**	**..t**	...	...	...	...	...	...	**A.A**	...	**G.G**	...	...	**G.T**	**C..**	...	**A..**	...	...	...	...	...
SalI: nt2542	...	...	...	...	...	**..t**	**..t**	...	...	...	...	...	...	**A.A**	...	**G.G**	...	...	**G.T**	**C..**	...	**A..**	...	...	...	...	...
AA 848	-	-	-	-	-	-	-	-	-	H	-	-	-	K	-	E	-	-	D	Q	-	K	-	-	-	-	-

Line 8

→ sV2 polymorphic subfragment

AA 858	K	T	K	I	N	Q	N	K	C	D	E	L	D	L	L	F	N	V	Q	N	N	L	P	A	M	Y	S	I	Y
Belem: nt 2572	**AAA**	**ACC**	**AAA**	**ATT**	**AAT**	**CAA**	**AAC**	**AAA**	**TGC**	**GAT**	**GAA**	**TTG**	**GAC**	**CTC**	**CTA**	**TTC**	**AAT**	**GTC**	**CAG**	**AAC**	**AAC**	**TTG**	**CCA**	**GCC**	**ATG**	**TAC**	**TCC**	**ATA**	**TAT**
Mx82	...	...	...	...	...	...	...	...	...	...	...	...	...	...	...	...	...	...	...	...	...	...	...	...	...	...	...	...	...
Mx96	**C..**	**.A.**	...	...	**..G**	**G..**	**..t**	...	...	...	**A..**	...	...	...	...	...	...	...	...	...	...	...	...	...	...	...	...	...	...
Mx30	**C..**	**.A.**	...	...	**..G**	**G..**	**..t**	...	...	...	...	...	...	...	...	...	...	...	...	...	...	...	...	...	...	...	...	...	...
MX16	**C..**	**.A.**	...	...	**..C**	**G..**	**..t**	...	...	...	...	...	...	...	...	...	...	...	...	...	...	...	...	...	...	...	...	...	...
MX14	**C..**	**.A.**	...	...	**..G**	**G..**	**..t**	...	...	...	...	...	...	...	...	...	...	...	...	...	...	...	...	...	...	...	...	...	...
MX97	**C..**	**.A.**	...	...	**..G**	**G..**	**..t**	...	...	**..c**	...	...	...	...	...	...	...	...	...	...	...	...	...	...	...	...	...	...	...
MX12	**C..**	**.A.**	...	...	**..G**	**G..**	**..t**	...	...	...	...	...	...	...	...	...	...	...	...	...	...	...	...	...	...	...	...	...	...
MX28	**C..**	**.A.**	...	...	**..G**	**G..**	**..t**	...	...	...	...	...	...	...	...	...	...	...	...	...	...	...	...	...	...	...	**C..**	...	...
MX98	**C..**	**.A.**	...	...	**..G**	**G..**	**..t**	...	...	...	...	...	...	...	...	...	...	...	...	...	...	...	...	...	...	...	...	...	...
MX27	**C..**	**.A.**	...	...	**..G**	**G..**	**..t**	...	...	...	...	...	...	...	...	...	...	...	...	...	...	...	...	...	...	...	...	...	...
MX99	**C..**	**.A.**	...	...	**..G**	**G..**	**..t**	...	...	...	...	...	...	...	...	...	...	...	...	...	...	...	...	...	...	...	...	...	...
MX100	**C..**	**.A.**	...	...	**..G**	**G..**	**..t**	...	...	...	...	...	...	...	...	...	...	...	...	...	...	...	...	...	...	...	...	...	...
MX9	**C..**	**.A.**	...	...	**..G**	**G.G**	**..t**	...	...	...	...	...	...	...	...	...	...	...	...	...	...	...	...	...	...	...	...	...	...
MX83	**C..**	**.A.**	...	...	**..G**	**G..**	**..t**	...	...	...	...	...	...	...	...	...	...	...	...	...	...	...	...	...	...	...	...	...	...
Sal I: nt 2623	**C..**	**.A.**	...	...	**..C**	**G..**	**.CT**	...	...	...	...	...	...	...	...	...	...	...	...	...	...	...	...	...	...	...	...	...	...
AA 875	Q	N	-	-	K	E	T	-	-	-	K	-	-	-	-	-	-	-	-	-	-	-	-	-	-	-	P	-	-

Line 9

```
                   → sV2 polymorphic subfragment    |→ 3´C (24 nucleotides)
        AA 887   D   S   M   S   N   E   L   Q   N   L   Y   I   E   L   Y   Q   K   903
Belem: nt 2659  GAC TCC ATG AGC AAC GAG CTG CAG AAT CTT TAC ATT GAG CTG TAC CAG AAG 2709

Mx83            ... ... ... ... ... ... ... ... ... ... ... ... ... ... ... ... .G.

Mx96            ... ..t ... ... ... ..a ... ... ..c ... ... ... ... ... ... ... .G.
Mx30            ... ..t ... ... ... ..a ... ... ..c ... ... ... ... ... ... ... .G.
MX16            ... ..t ... ... ... ..a ... ... ... ... ... ... ... ... ... ... .G.
MX14            ... ..t ... ... ... ..a ... ... ..c ... ... ... ... ... ... ... .G.
MX97            ... ..t ... ... ... ..a ... ... ..c ... ... ... ... ... ... ... .G.
MX12            ... ..t ... ... ... ..a ... ... ..c ... ... ... ... ... ... ... .G.
MX28            ... ..t ... ... ... ..a ... ... ..c ... ... ... ... ... ... ... .G.
MX98            ... ..t ... ... ... ..a ... ... ..c ... ... ... ... ... ... ... .G.
MX27            ... ... ... ... ... ..a ... ... ..c ... ... ... ... ... ... ... .G.
MX99            ... ..t ... ... ... ..a ... ... ..c ... ... ... ... ... ... ... .G.
MX100           ... ..t ... ... ... ..a ... ... ..c ... ... ... ... ... ... ... .G.
MX9             ... ..t ... ... ... ..a ... ... ..c ... ... ... ... ... ... ... .G.
MX82            ... ..t ... ... ... ..a ... ... ..c ... ... ... ... ... ... ... .G.

                ... ..t ... ... ... ..a ... ... ..c ... ... ... ... ... ... ... ... 2760
SalI: nt 2710
      AA 904     -   -   -   -   -   -   -   -   -   -   -   -   -   -   -   -   R   920
```

The nucleotide and amino acid sequence of Belem (AF435594)and Sal I (XM_001614792.1) strains are used as references; Mx, Mexican isolate; nt, nucleotide; nucleotide subfragments 5´C, M and 3´C were partially conserved and sV1 and sV2 were polymorphic.
AA, one letter amino acid code
Non-synonymous Nucleotide changes are indicated by capital letters
synonymous nucleotide changes are indicated by small case letters
dashes in AA indicate no amino acid change
